# Delayed Onset and Prolonged ECT-Related Delirium

**DOI:** 10.1155/2013/840425

**Published:** 2013-09-03

**Authors:** Sameer Hassamal, Ananda Pandurangi, Vasu Venkatachalam, James Levenson

**Affiliations:** ^1^Department of Psychiatry, Virginia Commonwealth University, P.O. Box 980710, Richmond, VA 23298, USA; ^2^Department of Psychiatry, Virginia Commonwealth University, P.O. Box 980268, Richmond, VA 23298, USA

## Abstract

Electroconvulsive therapy (ECT) is effective in the treatment of depression. Delayed post-ECT delirium is rare but can occur in a small subset of patients with risk factors and in most cases resolves with the use of psychotropic medications. We report a unique presentation of a patient who developed a delayed post-ECT delirium with fecal incontinence that commenced 24 hours after the administration of ECT. The condition resolved spontaneously after 48 hours without the use of psychotropic medications.

## 1. Introduction

ECT is an important treatment for severe depression including medication refractory depressive symptoms, mania, schizophrenia, and catatonia [1]. However, in the United States, ECT is most commonly used for the treatment of unipolar depression after multiple trials of psychotropic medications have failed to achieve either an adequate response or remission [2]. Although the exact mechanism of action is unknown, a variety of theories postulate that ECT increases monoamine neurotransmitter availability, induces a release of hypothalamic or pituitary hormones, and increases neurogenesis or synaptogenesis [3–5]. ECT has also been shown to have anticonvulsant properties [6]. 

Cardiovascular complications are the most common causes of morbidity and mortality following ECT [7, 8]. Other complications include headaches, muscle pain, fatigue, and nausea [9, 10]. Anterograde and retrograde amnesia are common ECT-related cognitive complaints [8]. Typically, anterograde amnesia resolves within a few weeks whereas retrograde amnesia is more persistent [8]. Both types of amnesia are more common with bilateral stimulation, high-intensity electrical dosage, and sinus waveform stimulus [11–13]. Mild confusion is reported in about 40% of cases; however, delirium is common following the first seizure or in those with predisposing brain pathology such as white matter lesions [9, 14, 15]. Additionally, patients taking lithium or dopaminergic medications concurrently with ECT are at an increased risk of delirium [16].

We present the case of a highly functional 41-year-old Caucasian woman who developed a severe and prolonged ECT-related delirium.

## 2. Case Presentation

Ms. XX, a 41-year-old Caucasian woman with no known medical illnesses was diagnosed with unipolar major depressive disorder at the age of 27 years. The current episode was her 3rd, lasted over 4 months, and had only partially responded to psychotropic medications including the combination of lithium carbonate 300 mg twice a day, mirtazapine 15 mg daily, and venlafaxine XR 450 mg daily. Ms. XX had also failed treatment with bupropion and augmentation with methylphenidate and aripiprazole. She scored 31 on the Montgomery-Åsberg Depression Rating Scale (MADRS) indicating moderate to severe depression. ECT was considered since she had failed at least four trials of antidepressants along with augmentation strategies and had become impaired to the point she could not function at work. On the week of ECT, mirtazapine was discontinued, and venlafaxine XR and lithium carbonate were tapered to 225 mg daily and 300 mg daily, respectively. 

Detailed neurological and physical examination before ECT did not reveal any abnormality. Baseline investigations including a complete blood count and comprehensive metabolic panel of electrolytes, glucose, liver, and renal functions were within normal limits. Electrocardiogram (EKG) was normal. Ms. XX scored 30/30 on a Mini Mental Status Examination (MMSE) indicating no cognitive deficits. Head computed tomography (CT) and Electroencephalography (EEG) were not performed. 

Right unilateral brief pulse treatment at 25% was initiated three times weekly using the Thymatron System IV. Prior to each ECT session, etomidate 16 mg and succinylcholine 100 mg were administered (etomidate was chosen due to a nation-wide shortage of methohexital which is the preferred anesthetic at our institution). The seizure response was 76 and 48 seconds after the first and second treatments, respectively. After the second ECT session, Ms. XX developed a mild agitated confusional state, which resolved immediately after receiving 2 mg of intravenous midazolam. There was no persistence of agitation or confusion over the next 2 days. On the 3rd ECT session, the energy was increased to 50% to obtain a robust seizure response. After the third ECT session, Ms. XX again developed an agitated state, which also resolved immediately after 2 mg of intravenous midazolam. No other cognitive side effects were detected on clinical interview, and the patient was discharged home in a fully oriented state. 

As per the report of her husband, she rested at home and was not confused or agitated the rest of the day. However, 24 hours after the third ECT session, she developed a delirium consisting of confusion, disorientation, markedly increased speech latency time, and fecal incontinence. The on-call psychiatrist was contacted who recommended a visit to the emergency room, but the husband decided to wait until Monday to see the ECT team. On Monday, 72 hours after the last ECT treatment, Ms. XX presented to the ECT psychiatrist. Examination confirmed a severe confusional and disoriented state with minimal to no responses to questions. There was no evidence of any restlessness or perceptual disturbances such as illusions or hallucinations. She was ambulatory without ataxia and with no evidence of any focal deficits on neurological examination. 

She was admitted to the inpatient psychiatric unit for safety and further assessment. Investigations including serum electrolytes, renal and liver functions, blood sugar, and head CT scan were normal. The lithium level was <0.3 mEq/L, and the RBC-lithium was <0.10 mEq/L. EEG showed intermittent disorganization and slowing over both frontal and temporal regions suggestive of moderate bihemispheric dysfunction, which can be seen with both metabolic and epileptic encephalopathies. The patient's cognition started to improve in the hospital 72 hours after the last ECT and 48 hours after the onset of the confusional state, without the use of psychotropic medications. Cognition and behavior were assessed to be at or near baseline, and she was discharged home after 24 hours of observation. Interestingly, her depression had significantly improved as well. 

## 3. Discussion

The etiology of the patient's delirium is unclear. Based on our observations, it appears as though ECT induced a delayed and prolonged confusional state that resolved spontaneously. However, multiple factors may have played a role. The condition could have been secondary to the ECT; the combination of ECT, lithium, and venlafaxine; an atypical reaction to midazolam; or some combination of the above. It is not clear why this occurred only after the third ECT rather than the first or second treatments. On the other hand, the agitation episodes after the first and second treatments could have been harbingers of the delirium to come. A new neurological or metabolic delirium onset was considered unlikely as the patient improved spontaneously and maintained an improved cognitive (and mood) status at 2-week followup. 

A Medline search revealed two recent cases describing a similar delayed onset delirium after ECT. Both of these cases described this presentation as a delirium [17, 18]. An interictal delirium usually occurs after a prolonged period of disorientation following ECT or can even occur independently of the post-ictal state [18, 19]. The most common risk factors are older age, subcortical and basal ganglia lesions, heart disease, Parkinson's disease, and Alzheimer's disease [20, 21]. Our patient did not have any of these risk factors. Two other subtypes of delirium after ECT have been described. The most common subtype is an acute post-ictal disorientation characterized by a confusional state immediately after ECT typically self-resolving in an hour [18, 22]. The third is a post-ECT delirium highlighted by hyperactivity that usually begins a few minutes after the seizure induced by ECT [17, 18, 23]. Most post-ECT delirium cases last less than an hour, tend to occur during the initial ECT treatments especially in those switched from unilateral to bilateral treatment, and usually resolve spontaneously or after the administration of diazepam, midazolam, barbituratesm, or droperidol [16, 18, 24]. Our patient's presentation did not fit the above scenarios. Devanand and colleagues found that the degree of pretreatment agitation, anesthetic or succinylcholine dose, number of ECT's received, or mean seizure duration did not predict those who would develop post-ictal excitement [24]. 

There is conflicting data regarding the safety of lithium and ECT. Some studies have shown a neurotoxic interaction between lithium and ECT, and others have shown no significant increase in the incidence of complications [25–27]. Although both lithium and ECT can independently produce a delirium, Penney et al. found that concurrent lithium and ECT is associated with an increased incidence of severe or prolonged confusion [26]. The administration of lithium in close temporal relationship to ECT was associated with an increased risk of a confusional state [26]. The serum lithium level, diagnosis, number, and laterality of ECT treatments were not associated with the development of an acute confusional state [26]. The onset and duration of this neurotoxic interaction are quite variable and may begin from hours to days after ECT and typically resolve over days to months [28, 29]. This synergistic neurotoxic effect has been documented at lithium levels as low as 0.37 mEq/L [30, 31]. El-Mallakh suggested that the neurotoxic interaction is most likely secondary to ECT-induced intracellular lithium toxicity at normal serum lithium levels [32]. Additionally, ECT increases the permeability of the blood brain barrier, which in turn may lead to a faster entry of lithium ions [33, 34].

The concomitant use of antidepressants and ECT is controversial in terms of efficacy and adverse side effects [35]. However, due to concern about adverse side effects, the American Psychiatric Association Task Force on ECT discourages the concomitant use of antidepressants and ECT [36]. Concomitant use of venlafaxine and ECT has been found to improve clinical outcomes but may worsen cognitive side effects [35]. There have been no case reports of a prolonged complicated delirium after the concomitant use of venlafaxine and ECT. 

There has been at least one case report documenting serotonin syndrome during treatment with lithium and venlafaxine in spite of normal lithium levels [37]. However, our patient's delirium occurred at lower doses of both drugs than when she has been prior to ECT, making the presence of serotonin syndrome is unlikely. However, neuroleptics were stopped weeks before ECT administration. In our patient and consistent with our own ECT preparation guidelines, a precautionary decrease in lithium dose by 50% was implemented just prior to commencement of the ECT course. If lithium played any role in the subsequent delayed delirium after the 3rd ECT, obviously the decrease in dose did not help. 

Midazolam is an attractive agent for managing post-ictal hyperactivity after ECT due to its rapid clinical effect, rapid elimination half-life of 2 hours, and low toxicity profile [38, 39]. The mean dose is usually 1.5 ± 0.9 mg, although repeat doses may be necessary in some patients [40]. The onset of action is usually less than 90 seconds and the recovery time is usually 45 ± 30 minutes but can vary substantially [40]. In a clinical trial of 21 patients receiving midazolam for post-ictal hyperactivity, midazolam was found to be safe in all patients and effectively treated delirium in 20 of the 21 patients [40]. Liston did not report any cases of a prolonged complicated delirium after administration of midazolam. However, midazolam can induce a paradoxical reaction with agitation and confusion in less than 1% of all patients receiving midazolam [41]. This paradoxical reaction occurs most commonly in patients with psychiatric or personality disorders [42–44]. In a clinical trial of 58 patients receiving midazolam for lower body surgery, Weinbroum and colleagues found that the midazolam-induced paradoxical reaction begins 45–210 minutes after sedation is started and can be reversed with flumazenil [45].

What is unique about our case is the *de novo* onset of delirium more than 24 hours after the ECT and treatment of a post-ictal confusional state with midazolam. Although there are limited case reports describing delirium after ECT, none of them describe fecal incontinence. Additionally, the delirium resolved spontaneously without psychotropic medications. The unique presentation as well as the spontaneous resolution of the delirium may represent a unique subtype of interictal delirium after ECT. In our case, the combination of lithium and venlafaxine as well as midazolam may have contributed to this interictal delirium. 

In our case, the delirium resolved after 48 hours and did not require the use of psychotropic medications. Some case reports of interictal delirium have persisted and required antipsychotics, benzodiazepines, and donepezil [18]. 

## 4. Conclusion

We present a case of a 41-year-old otherwise healthy individual with a medication unresponsive unipolar depression, who developed a delayed onset delirium after the 3rd ECT that resolved spontaneously after 48 hours, with a concurrent improvement in the depression. No specific etiology could be established. Recommendations regarding the use of specific psychotropic medications such as lithium during ECT need to be further investigated due to case reports of adverse side effects. Risk factors, treatment guidelines, and clinical outcomes regarding a prolonged interictal delirium after ECT need to be further elucidated.
